# Cesarean Section Performed by a Cow

**Published:** 1907-05

**Authors:** 


### CESAREAN SECTION PERFORMED BY A COW.

Mrs. W. E., aged 31, pregnant at full term with her third child, was standing in the road about sixty feet from the house, watching her husband unload hay from a wagon. A vicious two-year-old heifer charged her and inserted a very sharp horn into the abdomen near the anterior superior spinous process of the ilium. It penetrated the abdominal wall, uterus and membranes, but escaped the child. the point of the horn then emerged again just to one side and below the umbilicus. The result was two transverse rents, the upper one about five inches long and the other clear across the abdomen. The child was delivered through the larger opening and fell to the road, where it was picked up by the father. I made an eight-mile trip and arrived about twenty minutes after the mother's death from hemorrhage. The uterus was outside the body, torn clear across and inverted, with afterbirth and membranes, still attached. The child is still alive and doing well. I have found histories of several similar cases in Gould and Pyle's work on medical curiosities.—W. B. Morse, M. D., Journal A. M. A.

